# Repeatability and reliability of kinetic and temporospatial gait parameters measured with a pressure-sensitive treadmill in healthy cats

**DOI:** 10.3389/fvets.2025.1707477

**Published:** 2025-12-17

**Authors:** Dorothea M. Weißig, Britta Dobenecker, Yury Zablotski, Susanne K. Lauer

**Affiliations:** 1 LMU Small Animal Clinic, Centre for Clinical Veterinary Medicine, Ludwig-Maximilians-University, Munich, Germany; 2 Diagnostic Centre for Small Animals - Day Clinic, Dresden, Germany; 3 Department of Veterinary Sciences, Ludwig-Maximilians-University, Munich, Germany

**Keywords:** gait analysis, pressure-sensitive treadmill, locomotion, temporospatial parameter, kinetic gait, feline, cat

## Abstract

**Objectives:**

Interest in feline kinetic gait variables has increased substantially. This study aimed to assess repeatability and intersession reliability of kinetic gait analysis in healthy cats using a pressure-sensitive treadmill.

**Methods:**

Healthy client-owned cats (*n* = 9) and cats housed at the cattery of the Chair of Animal Nutrition and Dietetics (*n* = 5), without orthopedic abnormalities based on history, examination, and subjective gait analysis, were enrolled. Cats (mean body weight: 5.2 ± 0.9 kg, age: 4.1 ± 1.7 years) were acclimated to a pressure-sensitive treadmill system (FDM-T-CanidGait®, zebris Medical GmbH, Isny, Germany). Treadmill velocity was adjusted individually for comfortable walking. For data acquisition, cats were placed on the treadmill and repositioned if they jumped off (total duration: 10 min, 5 × 2 min). The first five sequences with six valid gait cycles were selected. Data acquisition was repeated after 2 weeks at the same speed. Average maximal pressure, loaded paw surface, step and stride length, step width, stance and swing phase percentages, hind reach, step-stride ratio, and symmetry indices were calculated. Repeatability was assessed with linear mixed-effects models and intersession reliability with intraclass correlation coefficients (ICCs). Reliability was categorized as excellent, good, moderate, or poor, with statistical significance set at a *p*-value of < 0.05.

**Results:**

Most parameters did not differ significantly between time points, except for the average maximal pressure of the left hindlimb (*p* = 0.037) and hind reach of both hindlimbs (*p* ≤ 0.001). ICCs demonstrated good to excellent reliability (0.885–0.988) for all variables, except for symmetry indices (moderate reliability; forelimbs: 0.547, hindlimbs: 0.676).

**Conclusion and relevance:**

A pressure-sensitive treadmill provides repeatable feline gait measurements with good to excellent intersession reliability for most variables and moderate reliability for symmetry indices, offering a reference for future studies. Further studies involving larger cohorts are needed to confirm these results and support broader clinical application.

## 1 Introduction

During the past two decades, the number of domestic cats in Germany has increased significantly from 6.8 to 15.7 million (2000–2023) (1). In parallel, interest in feline gait and its analysis has also grown. The number of publications on feline gait analysis has more than doubled when comparing the periods 2000–2002 and 2022–2024 (online database searches: PubMed and Google Scholar, search terms: “cat” OR “feline” AND “gait” OR “locomotion” AND “analysis”).

Subjective visual lameness evaluation is known to have high intra- and inter-observer variability in dogs (2, 3) and should also be interpreted with caution in cats. Therefore, objective kinetic and kinematic gait analysis is essential to improve understanding of normal feline gait and to assess the effects of surgical, dietary, or medical interventions in cats with musculoskeletal disorders.

Compared to dogs, objective gait analysis in cats poses greater challenges, as they are often reluctant to walk or may adopt a crouched gait in clinical settings (4–6). However, cat-friendly handling techniques and sufficient acclimatization time can markedly facilitate kinetic gait analysis in cats (5, 6). A recent feline study also introduced deep learning-based kinematic gait analysis that eliminates the need for reflective markers, reducing stress and gait alterations during marker placement in cats (7). The availability of user-friendly pressure-sensitive walkways has increased the feasibility of performing kinetic gait analysis in clinical settings, extending its application beyond research environments. However, as most cats are not leash-trained, controlling de- and acceleration, walking speed, and direction is difficult in cats, complicating feline gait analysis on pressure walkways (8–11).

Although kinetic gait analysis is still rarely applied in clinical practice, its use is increasing in research, particularly in studies on cranial cruciate ligament injuries (12), osteoarthritis (13, 14), outcomes of femoral head and neck ostectomy, total hip replacement (15, 16), and monitoring the post-onychectomy recovery (17).

Pressure-sensitive treadmills and treadmill systems with integrated force platforms offer advantages, such as velocity control and potentially a more consistent linear gait pattern compared to walkway or floor-integrated force platform systems (18). This is crucial, as velocity changes may affect ground reaction forces and temporospatial parameters, mimicking lameness or musculoskeletal abnormalities (2, 19). Moreover, the unlimited “walkway” of a treadmill enables analysis of multiple continuous gait cycles, avoiding disruptions common on floor-based systems (2, 20). This continuity also allows for the observation of fatigue-related asymmetries, as there is no muscular recovery phase between trials (21).

Initial investigations of feline kinetic gait analysis using instrumented treadmill systems have predominantly been confined to experimental non-clinical settings (22–25). To the best of the authors’ knowledge, systematic analyses of ground reaction forces in feline treadmill locomotion have not yet been conducted (26–29).

The objective of this prospective study was to evaluate the reproducibility and reliability of kinetic gait analysis using a commercially available pressure-sensitive treadmill system (CanidGait®, zebris Medical GmbH, Isny, Germany) and to establish baseline reference values for healthy cats.

## 2 Materials and methods

### 2.1 Study population

This prospective study included healthy client-owned cats (*n* = 9) and cats housed at the cattery of the Chair of Animal Nutrition and Dietetics (*n* = 5) without orthopedic or neurological abnormalities based on history, general and orthopedic examination, and subjective gait analysis. Inclusion criteria were a minimal body weight of 4 kg and a body condition score (BCS) between 4 and 6 on a 9-point scale (30). All cats were required to walk freely, voluntarily, and comfortably on the treadmill to qualify for inclusion. Exclusion criteria comprised the presence of infectious or immunological diseases, clinically relevant cardiac conditions, or medication with analgesics or non-steroidal anti-inflammatory drugs within 14 days prior to study participation.

### 2.2 Kinetic gait analysis

Kinetic gait analysis was performed using a pressure-sensitive treadmill system (FDM-T-CanidGait®; sensitivity is 0.5 N/cm^2^; Animal Analysis Suite RC-2.3.28; zebris Medical GmbH, Isny, Germany) with a sampling frequency of 100 Hz. Two video cameras (WinFDM Software v1.2.2; zebris Medical GmbH, Isny, Germany) synchronized with the treadmill were positioned behind and to the left of the treadmill to document the trials. Prior to data acquisition, each cat was individually acclimated to the testing environment, treadmill, and handlers, and was allowed to roam freely for up to 15 min. During this period, cats were encouraged to explore the room and the treadmill while it was switched off, and they were gradually exposed to the sounds and motion of the treadmill at low speeds. Handlers interacted calmly using toys or treats until the cat displayed a relaxed posture, steady exploration, and voluntary approach toward the treadmill and handlers, which were taken as signs of habituation. Testing conditions differed between client-owned cats and those housed in the cattery of the Chair of Animal Nutrition and Dietetics. Client-owned cats were examined in a 5 × 10 m room at the Animal Mobility Centre of the LMU Small Animal Clinic, whereas the cats at the Chair of Animal Nutrition and Dietetics were assessed in a 3 × 8 m room.

**Figure 1:**
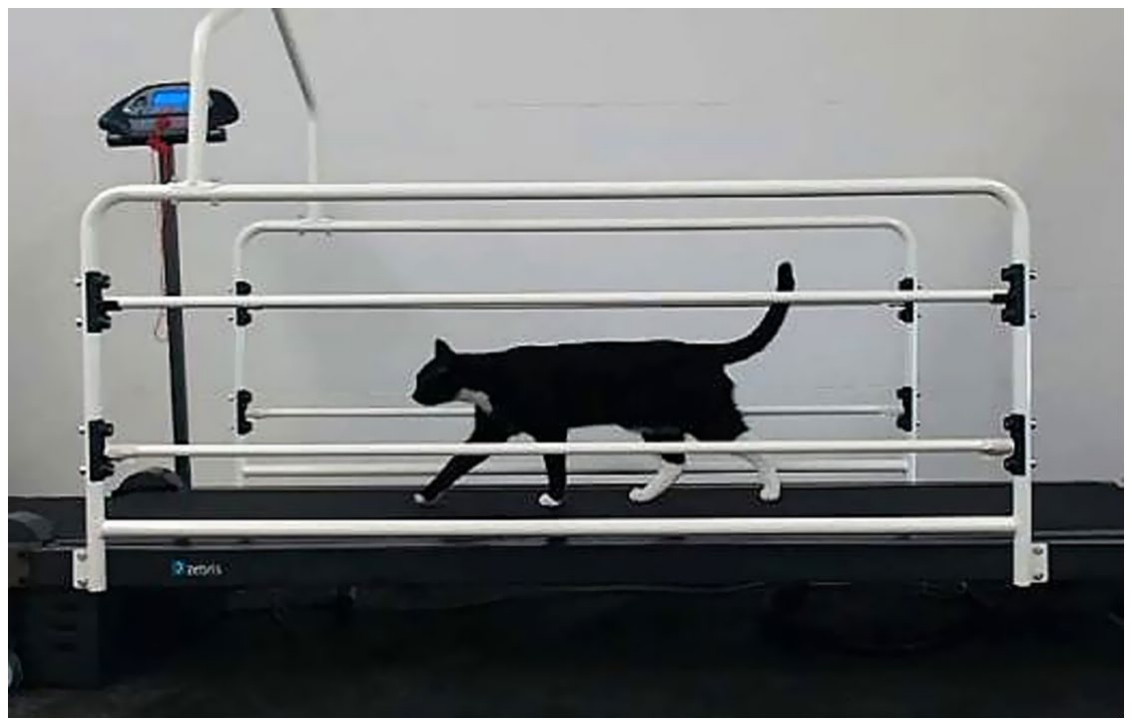
Cat walking on a pressure-sensitive treadmill system (FDM-T-CanidGait®; sensitivity 0.5 N/cm2; Animal Analysis Suite RC-2.3.28; Zebris, Isny, Germany).

To initiate the trial, a handler placed the cat on a pedestal at the rear of the treadmill, prompting it to cross the motionless treadmill. A second caregiver, positioned behind the pedestal at the front of the treadmill, encouraged the cat to walk over the treadmill using verbal cues, toys, clickers, or food, depending on each cat’s preference. If the cat was comfortable walking across the motionless treadmill, it was then placed directly onto the moving treadmill set at lower velocity. If the cat was unwilling to cross the motionless treadmill, it was placed directly onto the moving treadmill. The treadmill speed was then gradually increased in 0.1 km/h increments until the cat reached a steady-state walking pattern (Figure 1). Steady-state walking was defined as continuous locomotion at a constant treadmill speed, characterized by uniform stride patterns and the absence of interruptions such as acceleration, deceleration, or stopping. A 2-min trial was conducted once a steady gait was achieved. If the cat jumped off during the trial, it was gently returned to the running treadmill, provided it remained comfortable and willing. Each cat completed five trials, contingent on its continued comfort and cooperation. Acclimation time was limited to a maximum of 30 min for each time point, and the total evaluation time did not exceed 60 min. Data acquisition was repeated after 2 weeks at the same velocity. Initial measurements of treadmill velocity were recorded at the first time point, and this velocity was subsequently used to assess treadmill walking at the second time point. Environmental conditions, including lighting and noise levels, were kept as constant as possible throughout the testing. Examinations were performed at normal room temperature under standard artificial lighting, and trials were repeated if unexpected noise or other disturbances occurred.

### 2.3 Data evaluation

All trial sequences were processed using the complementary Animal Analysis Suite RC 2.3.28 software (zebris Medical GmbH, Isny, Germany). Gait cycles were excluded from analysis if apparent de- or acceleration, head movements, or directional changes were observed during video analysis. Small movements of the ears and tail, however, were considered acceptable. A valid trial was defined as a gait sequence of six consecutive steps at each paw. The first five valid sequences were used to calculate the average maximal pressure (% body weight), loaded paw surface (cm^2^), step and stride length (cm), step width (cm), stance and swing phase percentages (%), hind reach (cm), step-to-stride ratio, symmetry indices, and cadence at the two time points. Symmetry indices (SI) of the average maximum pressure (AMP) were calculated for each cat using the following formula:


$$\mathrm{SI}=200*\frac{\left(\mathrm{AMP}[\text{right}]-\mathrm{AMP}[\text{left}]\right)}{(\mathrm{AMP}[\text{right}]+\mathrm{AMP}[\text{left}])\;}$$

The SI was calculated separately for the forelimbs and hindlimbs. A value of less than 9 was considered symmetrical (2).

### 2.4 Statistical analysis

Repeatability was evaluated using linear mixed-effect regression. Because of repeated measures, generalized linear mixed-effects models with the individual animal as a random effect were applied. Model assumptions were systematically assessed: (1) the normality of residuals with the Shapiro–Wilk normality test, (2) the homogeneity of variances between groups with Bartlett’s test, and (3) the heteroscedasticity (constancy of error variance) with the Breusch–Pagan test. If assumptions were fulfilled, generalized linear mixed effects models (R package - lmer) were applied; if violated, robust linear mixed effects models were applied (R package - robustlmm). Model assumptions were not met for the stance phase, swing phase, double stance, symmetry index, hind reach, and velocity. Linear and robust linear models were compared using the conditional coefficient of determination R2, the marginal coefficient of determination R2, the intraclass-correlation coefficients (ICCs), and root mean square error (RMSE) as performance quality indicators. The model with the most favorable overall fitting power was retained. Contrasts between the categories (time points or limbs) were evaluated after model-fitting using estimated marginal means (R package - emmeans) with Tukey *p*-value correction for multiple comparisons. Results are presented as means with 95% confidence intervals (95% CI), unless otherwise indicated. In addition, intraclass correlation coefficients (ICCs) were calculated to assess intersession reliability, providing a complementary, widely recognized metric to confirm the findings and to convey reliability intuitively. Reliability was categorized as excellent (ICC > 0.9), good (0.75–0.9), moderate (0.5–0.75), and poor (< 0.5) (31). Statistical significance was defined as a *p*-value of < 0.05. Depending on the data distribution, descriptive results are reported as mean ± standard deviation (SD) or as median with interquartile range (IQR). All analyses were performed using R (version 4.5.0, 2025-04-11; R Foundation for Statistical Computing, Vienna, Austria) and IBM SPSS Statistics (version 28; IBM Corp., Armonk, NY, USA).

## 3 Results

The study population comprised nine European Shorthair cats, two Norwegian Forest Cats, one Bengal, one domestic longhair, and one Maine Coon mix. Three were spayed females, two were intact males, and nine were neutered males. The cats had a mean age of 4.1 ± 1.7 years. Their mean body weight was 5.2 ± 0.9 kg, and the median body condition score was 5 (range 4–6) on a 9-point scale. The cats walked on the treadmill at a mean velocity of 2.4 ± 0.4 km/h.

**Figure 2:**
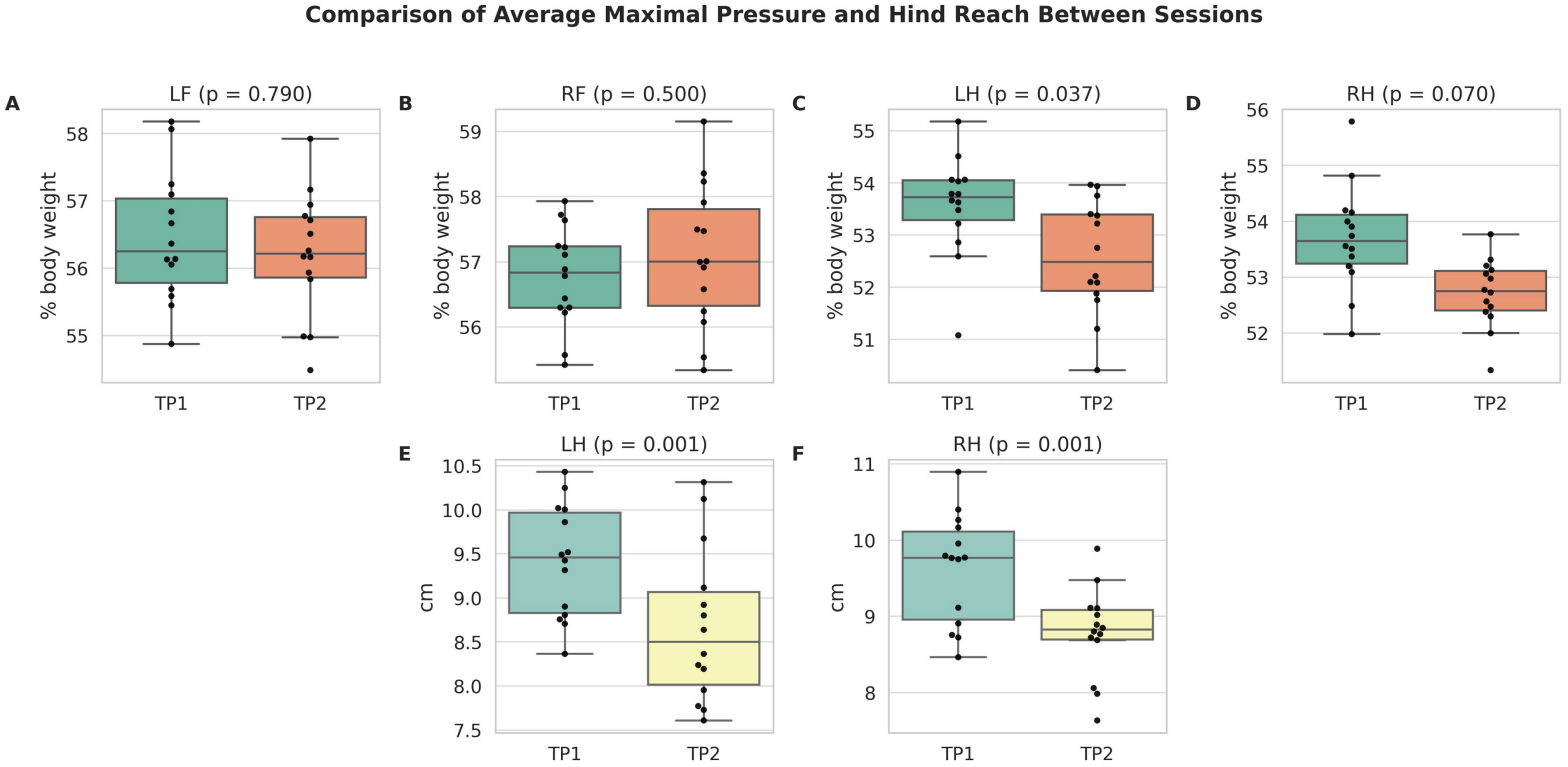
Comparison of average maximal pressure **(A–D)** and hind reach **(E,F)** between time point 1 (TP1) and 2 (TP2) for each limb. Boxplots show medians and interquartile ranges; black dots represent individual cats (*n* = 14 per session). *p*-values from mixed-effects models are shown above each pair. Only small differences were observed between time points, confirming high repeatability and intersession reliability.

Limb-dependent kinetic and temporospatial parameters at both time points are presented in Table 1. At time point 1, the average maximal pressure was 56.6% of body weight (95% CI, 54.4–58.8) in the left forelimb, 56.9% (95% CI, 54.7–59.1) in the right forelimb, 53.7% (95% CI, 51.5–55.9) in the left hindlimb, and 53.9% (95% CI, 51.7–56.1) in the right hindlimb. The mean loaded paw surface measured approximately 18–19 cm^2^ across all limbs. Step lengths ranged from 25.6 to 26.6 cm. The stance phase accounted for 59–63% of the gait cycle, while the swing phase accounted for 37–41%. The step-to-stride ratio remained close to 50% in all limbs. Limb-dependent parameters did not differ significantly between time points, except for the average maximal pressure of the left hindlimb (*p* = 0.037) and hind reach of both hindlimbs (*p* = 0.001; *p* < 0.001) (Figure 2).

**Table 1:** Comparison of kinetic and temporospatial parameters between time point 1 (TP1) and time point 2 (TP2).

Parameter	Limb	TP 1		TP2		Effect size		*p*-value
		Mean	95% CI	Mean	95% CI	Δ	95% CI	
Average loaded surface (cm^2^)	LF	17.7	16.6–18.8	17.8	16.7–18.8	−0.061	0.551–0.428	0.806
	RF	17.7	16.6–18.8	17.9	16.8–19.0	−0.164	−0.653–0.326	0.512
	LH	18.9	17.8–20.0	19.0	19.0–20.1	−0.164	−0.653–0.326	0.512
	RH	18.8	17.7–19.9	19.2	18.1–20.2	−0.368	−0.857–0.121	0.140
Average max. pressure (% BW)	LF	56.6	54.4–58.8	56.4	54.2–58.6	0.165	1.053–1.38	0.790
	RF	56.9	54.7–59.1	57.3	55.1–59.5	−0.418	−1.636–0.8	0.500
	LH	53.7	51.5–55.9	52.4	50.2–54.6	1.297	0.079–2.51	0.037
	RH	53.9	51.7–56.1	52.8	50.6–55.0	1.127	0.091–2.34	0.070
Step length (cm)	LF	25.6	24.3–26.9	26.6	24.3–26.9	- 0.044	−0.397–0.308	0.806
	RF	26.1	24.8–27.4	25.9	24.6–27.2	0.209	−0.144–0.561	0.246
	LH	25.8	24.5–27.1	25.6	24.3–26.9	0.257	−0.096–0.609	0.153
	RH	26.1	24.8–27.4	26.1	24.7–27.4	0.018	−0.335–0.37	0.922
Step-to-stride-ratio (%)	LF	49.4	49.0–49.7	49.6	49.3–50.0	−0.248	−0.744–0.248	0.327
	RF	50.5	50.1–50.8	50.3	49.9–50.6	0.209	−0.287–0.706	0.408
	LH	49.8	49.5–50.2	49.5	49.1–49.8	0.326	−0.17–0.822	0.198
	RH	50.4	50.0–50.7	50.6	50.2–50.9	−0.172	0.668–0.324	0.497
Stance phase (%)	LF	62.2	60.9–63.5	61.9	60.6–63.2	0.322	−0.192–0.835	0.220
	RF	62.8	61.5–64.1	62.7	61.5–64.0	0.014	−0.499–0.528	0.957
	LH	59.0	57.7–60.3	59.3	58.0–60.6	−0.294	−0.807–0.22	0.262
	RH	59.2	57.9–60.5	59.1	57.8–60.4	0.077	0.436–0.59	0.769
Swing phase (%)	LF	37.8	36.6–39.0	38.1	36.9–39.3	−0.318	−0.834–0.198	0.227
	RF	37.2	36.0–38.5	37.2	36.0–38.5	−0.018	−0.534–0.498	0.947
	LH	40.7	39.4–41.9	40.7	39.5–41.9	−0.028	−0.544–0.488	0.916
	RH	40.8	39.6–42.0	40.9	39.6–42.1	−0.289	−0.604–0.427	0.737
Hind reach (cm)	LH	9.45	7.27–11.6	8.59	6.41–10.8	0.859	0.331–1.39	0.001
	RH	9.59	7.41–11.8	8.56	6.38–10.7	1.028	0.5–1.56	<0.001

LF, left forelimb; RF, right forelimb; LH, left hindlimb; RH, right hindlimb; BW, body weight; Δ = estimated mean difference (TP1–TP2) obtained from the linear mixed (robust) model.

Values are expressed as means with 95% confidence intervals (CIs), estimated mean differences (TP1–TP2) with 95% CI, and *p*-values.

Temporospatial parameters not assigned to individual limbs at both time points are presented in Table 2. At time point 1, the mean stride length was 51.7 cm (95% CI, 49.2–54.4), and the mean velocity during data acquisition was 2.3–2.4 km/h. Step width was 3.2 cm in the forelimbs and 2.8 cm in the hindlimbs. Double stance constituted 25% of the gait cycle in the forelimbs and 18% in the hindlimbs. Symmetry indices were consistently below 9, indicating symmetrical gait across the study population. These parameters did not differ significantly between time points, except for the hindlimb symmetry index (*p* = 0.018).

**Table 2:** Comparison of temporospatial parameters between time point 1 (TP1) and time point 2 (TP2).

Parameter	TP 1		TP 2		Effect size		*p*-value
	Mean	95% CI	Mean	95% CI	Δ	95% CI	
Velocity (km/h)	2.4	2.2–2.6	2.3	2.1–2.6	0.03	−0.02–0.08	0.181
Stride length (cm)	51.8	49.2–54.4	51.6	49.0–54.2	0.19	−0.33–0.71	0.464
Step width front (cm)	3.16	2.75–3.57	3.29	2.88–3.70	−0.129	−0.507–0.248	0.501
Step width hind (cm)	2.82	2.41–3.23	2.80	2.40–3.21	0.014	−0.364–0.391	0.943
Double stance front (%)	25.0	22.4–27.5	24.7	22.1–27.3	0.262	−0.591–1.115	0.548
Double stance hind (%)	18.2	15.7–20.8	18.2	15.6–20.8	0.031	−0.822–0.884	0.944
SI front (avg. max. pressure)	0.711	−0.567–1.99	1.789	0.511–3.07	−1.08	−2.27–0.113	0.076
SI hind (avg. max. pressure)	0.446	−0.831–1.72	1.884	0.607–3.16	−1.44	−2.63–−0.247	0.018

SI, symmetry index; Δ = estimated mean difference (TP1–TP2) obtained from the linear mixed (robust) model.

Values are presented as means with 95% confidence intervals (CIs), estimated mean differences (TP1–TP2) with 95% CI, and *p*-values.

Intraclass correlation coefficients demonstrated good to excellent reliability for all parameters (0.885–0.988), except for the symmetry indices, which showed moderate reliability (ICC: forelimbs: 0.547; hindlimbs: 0.676) (Tables 3, 4).

**Table 3:** Reliability of kinetic and temporospatial parameters between both time points represented as intraclass correlation coefficients (ICCs) and 95% confidence intervals (CIs).

Parameter	LF		RF		LH		RH	
	ICC	95% CI	ICC	95% CI	ICC	95% CI	ICC	95% CI
Average loaded surface (cm^2^)	0.951	0.902–0.982	0.964	0.928–0.987	0.953	0.905–0.982	0.955	0.909–0.983
Average max. pressure (%BW)	0.969	0.937–0.988	0.968	0.937–0.988	0.968	0.936–0.988	0.969	0.939–0.988
Step length (cm)	0.986	0.972–0.995	0.983	0.965–0.993	0.988	0.976–0.995	0.976	0.952–0.991
Step-to-stride ratio (%)	0.891	0.780–0.959	0.885	0.769–0.957	0.933	0.865–0.975	0.932	0.864–0.974
Stance phase (%)	0.964	0.928–0.987	0.976	0.952–0.991	0.949	0.898–0.981	0.953	0.905–0.982
Swing phase (%)	0.964	0.928–0.987	0.976	0.952–0.991	0.957	0.914–0.984	0.953	0.905–0.982
Hind reach (cm)	-				0.974	0.948–0.990	0.979	0.957–0.992

LF, left forelimb; RF, right forelimb; LH, left hindlimb; RH, right hindlimb; BW, body weight.

**Table 4:** Reliability of kinetic and temporospatial parameters between both time points represented as intraclass correlation coefficients (ICCs) and 95% confidence intervals (CIs).

Parameter	ICC	95% CI
Stride length (cm)	0.988	0.976–0.996
Velocity (km/h)	0.980	0.961–0.993
Step width front (cm)	0.967	0.934–0.988
Step width hind (cm)	0.971	0.941–0.989
Double stance front (%)	0.978	0.955–0.992
Double stance hind (%)	0.969	0.937–0.988
SI front (avg. max. force)	0.547	0.082–0.830
SI hind (avg. max. force)	0.676	0.346–0.878

SI, symmetry index.

## 4 Discussion

This study investigated the acquisition of temporospatial parameters and gait patterns in healthy cats using a pressure-sensitive treadmill system. The hypothesis that the data would be repeatable and reliable was largely confirmed, with predominantly good or excellent reliability. These findings support treadmill-based gait analysis as an objective tool for feline gait assessment, particularly in cats reluctant to walk consistently on pressure walkways.

The statistically significant differences detected for the average maximal pressure of the left hindlimb and hind reach of both hindlimbs require cautious interpretation. While statistical significance was achieved, the magnitude of change was small and should not be assumed to indicate clinically meaningful alterations in gait. In recent treadmill studies in healthy dogs, mean absolute differences in peak vertical force between sessions were as low as 1.5 to 5.3% of body weight, with minimum detectable differences of up to 10% of body weight (32). These thresholds suggest that changes below this level likely reflect physiologic variability or habituation rather than true alterations in locomotor function. Reviews on objective gait analysis have similarly emphasized that statistical significance alone is insufficient for clinical interpretation and that effect sizes and minimum detectable differences provide a more meaningful assessment (33, 34). In the present study, the values remained within physiologic ranges, indicating that the observed changes are unlikely to be of clinical relevance. Habituation effects provide a plausible explanation for these findings. Short-term habituation has been documented in equine and human treadmill studies, with normalization of stance durations and kinematic patterns within minutes of exposure (35, 36). In dogs, repeated treadmill examinations revealed habituation effects for stance-phase duration and peak vertical force, manifesting as statistically significant but clinically minor changes between sessions (32). Comparable effects are plausible in cats, which often require longer acclimatization to novel environments and equipment (37). In unfamiliar environments, cats often exhibit stress-related behaviors such as crouching, hiding, or remaining motionless (4–6). These postural and behavioral adaptations can lead to acceleration or deceleration, altered paw loading, and inconsistent stride patterns, all of which may influence kinetic parameters and reduce measurement reliability. Adequate acclimatization is therefore essential to minimize behavioral artifacts and improve the repeatability and intersession reliability of gait data. The consistent reliability observed in the present study likely reflects the effectiveness of the standardized acclimatization protocol applied before data collection. The moderate intraclass correlation coefficients observed for symmetry indices also warrant cautious interpretation. Asymmetries of up to 6% have been described in clinically sound dogs during kinetic gait analysis (38–44). These findings indicate that moderate variability is not necessarily pathological but may reflect inherent biological variation. Symmetry indices should therefore not be interpreted in isolation but considered in conjunction with other kinetic and temporospatial parameters.

An important methodological advantage of treadmill-based gait analysis, in comparison to pressure-sensitive walkways or force plates, is the ability to maintain and precisely control velocity, thereby reducing variability associated with speed (20, 45). Given that variations in locomotor speed can markedly influence the accuracy and reliability of kinetic data, the ability to regulate velocity during treadmill-based assessments serves to reduce this source of variability (2, 18, 19). This control is particularly valuable in cats, where compliance and directional consistency are frequently challenging (5). Pressure-sensitive treadmill systems may therefore represent a robust adjunct to objective locomotor assessment and longitudinal monitoring of treatment outcomes (2, 39, 46–48).

A significant advantage of treadmill-based gait analysis is its capacity to capture changes in gait parameters related to fatigue or adaptation. This is facilitated by the “endless walkway” design, which enables uninterrupted observation of continuous locomotion (21). Unlike force plates or pressure walkways, the cat does not need to be repositioned after each pass, minimizing handling and manipulation and thereby reducing stress during gait assessment (4). Data acquisition and analysis using pressure-sensitive treadmills have also been shown to be considerably faster and more efficient than with pressure walkways, supporting their practical use in clinical and research contexts (20).

These technical and procedural advantages also translate into meaningful benefits for clinical application and research in feline locomotor disorders. The pressure-sensitive treadmill holds considerable translational value for both clinical and research applications. This method enables early detection of subtle gait asymmetries and provides objective quantification of locomotor recovery in cats with orthopedic or neurological disorders. In comparison with pressure walkways and force plates, treadmill-based gait analysis allows continuous steady-state assessment under controlled velocity, thereby improving measurement reliability (18). Although three-dimensional motion capture yields highly detailed kinematic information, the method requires the placement of skin markers and extended preparation time. Most cats do not tolerate markers well and benefit from minimal handling and short examination duration, which limits the clinical feasibility of this technique (7). Treadmill-based kinetic assessment, therefore, represents a practical and standardized tool for diagnostic evaluation and longitudinal follow-up in feline orthopedics and neurology.

A limitation of the present study is the absence of screening radiographs, as enrolled cats underwent only orthopedic examinations and subjective gait analysis. While radiographic imaging would have aided in excluding conditions such as osteoarthritis, when clinical signs are absent, sedation and full radiographic assessment in clinically healthy cats would have violated national animal welfare regulations. Consequently, osteoarthritis cannot be definitively ruled out in the study population, as it may not be detectable through physical examination and gait analysis alone (49–51). To mitigate this potential confounding factor, an age-based inclusion criterion was applied, given that the prevalence of osteoarthritis increases with advancing age (52, 53).

The study population also exhibited a sex bias, with a disproportionately high number of male cats. This imbalance was primarily due to fewer female cats meeting the inclusion criteria, as the minimum body weight threshold imposed by the sensitivity limits of the treadmill sensors excluded lighter individuals. Although this sex imbalance may represent a potential source of bias, prior studies using a pressure-sensitive walkway found no significant differences in temporospatial gait parameters between male and female crossbred cats, except for a longer stride length in males, which was attributed to their greater body size (54). Nevertheless, larger and more balanced samples are required to clarify whether sex influences gait characteristics in treadmill-based analyses. The overrepresentation of European Shorthair cats (9 of 14) in the present study may also limit the generalizability of the findings, although previous studies did not identify breed-related differences between Domestic Shorthair and Maine Coon cats (8).

The limited sample size reflects the exploratory nature of this study, which was intended to validate a novel measurement system for use with cats. Comparable pilot investigations in dogs and other species have likewise been conducted with small cohorts (18, 20). Considering the modest sample size, any generalization of these findings should be interpreted with caution. Future studies, including larger and more diverse cohorts, will be essential to establish reference intervals and to determine the clinical utility of treadmill-based gait analysis in cats with musculoskeletal and neurological diseases.

Walking on a treadmill may influence gait patterns and temporospatial parameters compared with overground locomotion, although published studies remain inconsistent. In dogs, treadmill walking has been associated with shorter stride length, increased stance time, and reduced swing time percentages (55), whereas another study reported only minimal differences, with mean deviations of less than 5% when compared to overground trotting (21). In mice, treadmill use increased stride frequency and decreased stride length but did not otherwise affect overall gait patterns compared to overground walking (56). In human participants, ground reaction forces and gait characteristics during treadmill walking were reported to be qualitatively and quantitatively comparable to overground walking, with no statistically significant differences (57–59). Such discrepancies may arise from species-specific locomotor adaptations, but also from methodological differences. The stationary body position relative to the environment during treadmill locomotion and mechanical inconsistencies in treadmill belt motion are likely sources of variability across studies (18). In a previous study comparing overground walking in dogs and cats, cats exhibited higher peak vertical forces during propulsion as well as greater propulsive force and hindlimb impulse (60). These findings suggest that cats may likewise show species-specific differences in gait parameters when walking on a treadmill compared to overground locomotion. Further research directly comparing treadmill and overground locomotion in cats is warranted to determine whether similar effects occur in this species.

In addition, a systematic assessment of feline acceptance of treadmill walking would be valuable, as acclimatization is critical for reliable measurements but was beyond the scope of the present study and would require a larger cohort.

## 5 Conclusion

In conclusion, this study demonstrates that a pressure-sensitive treadmill system provides repeatable measurements of feline gait, with good to excellent intersession reliability for most parameters. The statistically significant differences observed for a subset of variables were small, likely reflecting physiological variation or habituation, rather than clinically relevant changes. These findings establish a methodological foundation for future studies investigating gait alterations in cats with orthopedic and neurological disorders. Further studies on diseased feline populations are needed to confirm the clinical applicability and diagnostic value of this method.

## Data Availability

Restrictions apply to the availability of these data due to institutional and ethical regulations. Data are available from the corresponding author upon reasonable request and subject to approval.
